# Insight into Codon Utilization Pattern of Tumor Suppressor Gene EPB41L3 from Different Mammalian Species Indicates Dominant Role of Selection Force

**DOI:** 10.3390/cancers13112739

**Published:** 2021-06-01

**Authors:** Utsang Kumar, Rekha Khandia, Shailja Singhal, Nidhi Puranik, Meghna Tripathi, Atul Kumar Pateriya, Raju Khan, Talha Bin Emran, Kuldeep Dhama, Ashok Munjal, Taha Alqahtani, Ali M. Alqahtani

**Affiliations:** 1 Department of Biochemistry and Genetics, Barkatullah University, Bhopal 462026, India; utsangkr@gmail.com (U.K.); shailjasinghal95@gmail.com (S.S.); nidhipuranik30@gmail.com (N.P.); ashok.munjal@bubhopal.ac.in (A.M.); 2 ICAR-National Institute of High Security Animal Diseases, Bhopal 462043, India; tripathimeghna0107@gmail.com (M.T.); atul.pateriya@icar.gov.in (A.K.P.); 3 Microfluidics & MEMS Center, (MRS & CFC), CSIR-Advanced Materials and Processes Research Institute (AMPRI), Hoshangabad Road, Bhopal 462026, India; khan.raju@ampri.res.in; 4 Department of Pharmacy, BGC Trust University Bangladesh, Chittagong 4381, Bangladesh; talhabmb@bgctub.ac.bd; 5 Division of Pathology, Indian Veterinary Research Institute, Izatnagar, Bareilly 243122, India; 6 Department of Pharmacology, College of Pharmacy, King Khalid University, Abha 62529, Saudi Arabia; ttaha@kku.edu.sa (T.A.); amsfr@kku.edu.sa (A.M.A.)

**Keywords:** EPB41L3, codon usage bias, RSCU, GC contents, translational efficiency, mutational pressure, natural selection

## Abstract

**Simple Summary:**

The present study envisaged the codon usage pattern analysis of tumor suppressor gene EPB41L3 for the human, brown rat, domesticated cattle, and Sumatran orangutan. Most amino acids are coded by more than one synonymous codon, but they are used in a biased manner. The codon usage bias results from multiple factors like compositional properties, dinucleotide abundance, neutrality, parity, tRNA pool, etc. Understanding codon bias is central to fields as diverse as molecular evolution, gene expressivity, protein translation, and protein folding. This kind of studies is important to see the effects of various evolutionary forces on codon usage. The present study indicated that the selection force is dominant over other forces shaping codon usage in the envisaged organisms.

**Abstract:**

Uneven codon usage within genes as well as among genomes is a usual phenomenon across organisms. It plays a significant role in the translational efficiency and evolution of a particular gene. EPB41L3 is a tumor suppressor protein-coding gene, and in the present study, the pattern of codon usage was envisaged. The full-length sequences of the EPB41L3 gene for the human, brown rat, domesticated cattle, and Sumatran orangutan available at the NCBI were retrieved and utilized to analyze CUB patterns across the selected mammalian species. Compositional properties, dinucleotide abundance, and parity analysis showed the dominance of A and G whilst RSCU analysis indicated the dominance of G/C-ending codons. The neutrality plot plotted between GC12 and GC3 to determine the variation between the mutation pressure and natural selection indicated the dominance of selection pressure (R = 0.926; *p* < 0.00001) over the three codon positions across the gene. The result is in concordance with the codon adaptation index analysis and the ENc-GC3 plot analysis, as well as the translational selection index (P2). Overall selection pressure is the dominant pressure acting during the evolution of the EPB41L3 gene.

## 1. Introduction

Tumor suppressor genes (TSGs) are the genes that keep the check on the genes that are responsible for cell cycle progression. These genes further couple the DNA damage to the cell cycle, so that until DNA damage is repaired, the cell does not enter the cell division process. Further, if the damage is irreparable, these genes function in the direction of apoptosis. Inactivation of these genes removes/downregulates negative cues (inhibitory factors) of cell proliferation and contributes to unusual cell growth and division that leads to tumor development. The inhibitory molecules enciphered by most antioncogene (TSGs) inhibit cell division or endurance. Examples of tumor suppressor proteins are retinoblastoma protein (RB1), tumor protein p53 (TP53), B-cell leukemia/lymphoma 2 (BCL2), breast cancer 2 (BRCA2), etc. Inactivated Rb is involved in carcinomas of the bladder, breast, and lung. The TP53, INK4, and PTEN were found to be involved in lung cancer, prostate cancer, and melanoma. The adenomatous polyposis coli and MADR2/Smad2 genes are found altered in cases of colorectal cancers. Inactivation of the erythrocyte membrane protein band 4.1-like 3 (EPB41L3) gene through methylation is involved in breast cancer and renal clear cell carcinoma [1]. In the case of esophageal squamous cell carcinoma (ESCC), a cancerous tumor [2], EPB41L3 expression is reduced. In the case of EPB41L3 protein expression in ESCC host cells, inhibition of cellular proliferation, induction of apoptosis, and G2/M cell cycle arrest were observed via activation of caspase-3/8/9 and cyclin-dependent kinase 1/cyclin B1 signaling [3]. It alters the function of arginine N-methyltransferase proteins like PRMT3 and PRMT5.

EPB41L3 is also known as 4.1B or DAL1/4.1B and was found to disrupt the development of brain, breast, and lung tumor cells [4]. Loss of EPB41L3 has been found linked with the invasiveness and metastatic ability of non-small-cell lung cancer (NSCLC) causing carcinoma cells [5]. For the EPB41L3 gene, more than 20% methylation is called hypermethylation. The hypermethylation was found to be correlated with the advancement and continuation of NSCLC and an indicator of poor prognosis [6]. One of the distinctive aspects of the growth of brain tumors is the hypermethylation of TSGs [7]. The gene EPB41L3 is hypermethylated (29% hypermethylation) only in tumors, confirming its cancer-specific role [7]. It has been reported that EPB41L3 suppresses tumor metastasis and matrix metalloproteinase-2/9 activity in esophageal squamous cell carcinoma [3]. How EPB41L3 is associated with a tumor-suppressive role is poorly understood; however, it has a prognostic value. EPB41L3 is a protein-coding gene located on chromosome 18 (cytogenetic band 18p11.31). It consists of 1087 amino acids and has a molecular mass of 121 KDa.

The study of codon usage patterns in a particular gene or genome is essential for grasping the evolutionary features, identifying high-frequency, preferred, or underrepresented codon pairs, and to investigate the expression-linked codon usage patterns. Nucleotide composition plays a crucial role in shaping codon usage while the GC content is one of the key factors during the evolution of genomic structures [8]. It is well-known that most amino acids can be coded by synonymous codons that differ at only the third nucleotide positions due to the redundancy of the genetic code and thus are good indicators of the extent of synonymous codon usage bias. The change of the nucleotide at the third position of the codon does not change the amino acid; hence, a codon is changed, but its meaning in terms of the amino acid is not changed. The study of the third position of the codon also indicates the role of mutational pressure on codon usage bias. The study of the first and second nucleotide positions of the codon is also important since change of the base here leads to the change in the amino acid, too. These two positions (the first and the second positions of the codon) are indicative of selection forces. Many of the recent bioinformatics and experimental studies have revealed that the frequencies of the synonymous codon usage vary between different genes within and across organisms [9] due to evolutionary forces like mutational and selection pressure, etc. [10]. The recent advances in sequencing technologies and the availability of coding sequences (CDSs) in GenBank have empowered the comprehensive study of codon usage bias (CUB) indices of genes. The current study pivots on the comprehensive analysis of the codon utilization trends of the EPB41L3 genes in five mammalian species as codon usage by any organism is inconsistent in the midst of varying species [11], in-between kindred species and genes [12], and predominantly related to gene function [13]. The prime motive of the study was to computationally inspect the circumstances accountable for shaping codon usage in the EPB41L3 gene across the envisaged species through several methods including relative synonymous codon usage (RSCU), effective number of codons (ENc), nucleotide composition, and phylogenetic analysis.

Our study has provided a valuable acumen into the codon utilization trends of the EPB41L3 gene that is supposed to enhance our understanding of the tendencies in the preferential utilization of codons among the mammalian species as well as the significance of forces of evolution in shaping the codon usage.

## 2. Materials and Methods

### 2.1. Sequence Data Retrieval

A total of 34 complete transcripts of the EPB41L3 gene of five mammalian species accessible at the NCBI (http://www.ncbi.nlm.nih.gov/GenBank; accessed on 2 October 2020) were retrieved (FASTA sequences) and used. The dataset was created with special diligence; only those CDSs that have a start and a stop codon without undisclosed base throughout its length, excluding partial and intercalary stop codon CDSs, were taken into account. Finally, we retrieved 34 EPB41L3 transcripts that fulfil the criteria in five species of the class Mammalia, namely *Homo sapiens* (human), *Rattus norvegicus* (brown rat), *Bos taurus* (aurochs or domesticated cattle), *Mus musculus* (house mouse), and *Pongo abelii* (Sumatran orangutan), and cumulatively encompass 29,660 codons that were counted with the help of the Codon Usage Database (https://www.kazusa.or.jp/codon/countcodon.html; accessed on 2 October 2020). The full attributes of the datasets examined in the present work are provided in Table S1.

### 2.2. Nucleotide Content Analysis

The computed values for EPB41L3 transcripts of major compositional values such as overall GC (guanine and cytosine content) with GC1% (guanine and cytosine content at the first codon position), GC2% (guanine and cytosine content at the second codon position), and GC3% (guanine and cytosine content at the third codon position) along with the frequencies of A, T, G, and C including the nucleotides at the third codon position (A3%, T3%, G3%, and C3%) were analyzed. Apart from these, average GC3 contents were considered to study the effect of base compositional bias [14].

### 2.3. Relative Dinucleotide Abundance Analysis

Dinucleotide pair frequency has a significant role in codon usage as it is often used to establish an association between the dinucleotide bias and the CUB. Dinucleotide pair’s frequencies were calculated with the help of the EMBOSS explorer (http://emboss.bioinformatics.nl/cgi-bin/emboss/compseq; accessed on 3 October 2020). Dinucleotide frequency patterns help to understand both selection and mutational pressures [15]. The odds ratio (observed/expected dinucleotide frequency) below 0.78 is considered to signify underrepresentation while a ratio over 1.23 signifies overrepresentation [16,17].

### 2.4. Relative Synonymous Codon Usage Analysis

The RSCU values of all the 34 mRNA transcripts that included humans along with four other different mammalian species (Table 1) were calculated. The RSCU values were assessed using the CAIcal server [18]. Codons having the RSCU values less than 0.6 and more than 1.6 are known to be underrepresented and overrepresented respectively [19], whereas the values ranging in-between are considered unbiased.

### 2.5. Effective Number of Codons Analysis

Synonymous codons are referred to as two or more than two codons coding for a particular amino acid (except for Met and Trp). Wright (1990) [20] used ENc to identify the predilection in synonymous codon usage. ENc values range between 20 and 61 where ENc value 20 represents the absolute bias, i.e., for a particular amino acid, only one codon is used, while 61 indicates lack of bias, indicating identical use of all the synonymous codons, i.e., all the possible codons are used without any preference. However, the ENc value ≥35 represents a significant codon bias [21]. The ENc values are associated with translational efficiency of the respective genes [22] because of the utilization of the optimal codon among the synonymous codons [23]. The ENc values for all the transcript variants of the EPB41L3 gene across the species were calculated individually with the help of the CAIcal server (http://genomes.urv.es/CAIcal/; accessed on 2 October 2020).

### 2.6. Neutrality Plot

A plot of neutrality was used to determine the role of mutational force in the CUB against other evolutionary forces. The scatter plot was drawn between GC12 (Y-axis) and GC3 (X-axis). The regression line slope approaching 1 implies absolute neutrality and demonstrates a confined GC3 dispensation with the slope of the regression line reaching zero when mutational pressure overcomes the selection pressure during the evolutionary process [24].

### 2.7. Parity Rule Two Bias Plot Analysis

Ina PR2 bias plot (scatter diagram with four quadrants), the abscissa is the GC bias [G3/(G3 + C3)] at the third position of the base in a codon whilst the ordinate is the AT bias [A3/(A3 + T3)] at the third position of the base in a codon [25,26]. In the plot, the coordinate of the center is (0.5, 0.5), which demonstrates no biasness between mutation and selection rates, while the extent of deviation from the center indicates biasness [25,27].

### 2.8. Codon Adaptation Index Analysis

CAI is an effective method to predict a gene’s level of expression centered on how often a preferred codon is used. The value ranges from 0 to 1. CAI value 1 represents the highest relative adaptiveness. The higher the CAI value, the higher the gene expression potential and the CUB [15,28]. The CAI values were calculated for all the transcript variants of the EPB41L3 gene across the species individually with the help of the CAIcal server (http://genomes.urv.es/CAIcal/; accessed on 2 October 2020).

### 2.9. ENc-GC3 Plot

To explore the synonymous codon utilization trend of the EPB41L3 gene under the presence of evolutionary forces, the ENc-GC3 plot (where Enc—ordinate, GC3—abscissa) was drawn. The mutation is the primary factor influencing codon utilization as the resulting points collapse onto the anticipated curve, while the selection is the leading force in configuring codon utilization if the relevant points slip substantially below the anticipated curve [15,29].

The expected ENc values were calculated with the equation below where ‘*s*’ represents the frequency of GC3 codons [20].

$$ENc_{expected}=2+s+\frac{29}{s^{2}+\left(1-s^{2}\right)}$$

### 2.10. Translational Selection (P2)

P2 was determined to measure the efficacy of the codon–anticodon interaction and thus the gene’s expression level with the help of the following formula:

P2 = (WWC + SSU)/(WWY + SSY)

where W = A or T, S = C or G, and Y = C or T. A value of more than 0.5 reveals the bias towards translational selection [30].

### 2.11. Abundance Analysis of tRNA

For a single amino acid, different tRNA isotypes in various species bind to different codons. It is hypothesized that the most preferred codons are recognized by most abundant isoacceptor tRNAs, indicating the role of selection pressure [29]. The tRNA frequencies of each mammalian species were retrieved using GtRNAdb (genomic tRNA database).

## 3. Results

### 3.1. Nucleotide Composition in the EPB41L3 Gene Indicated G/C-Ending Codons Preference

**Figure 1:**
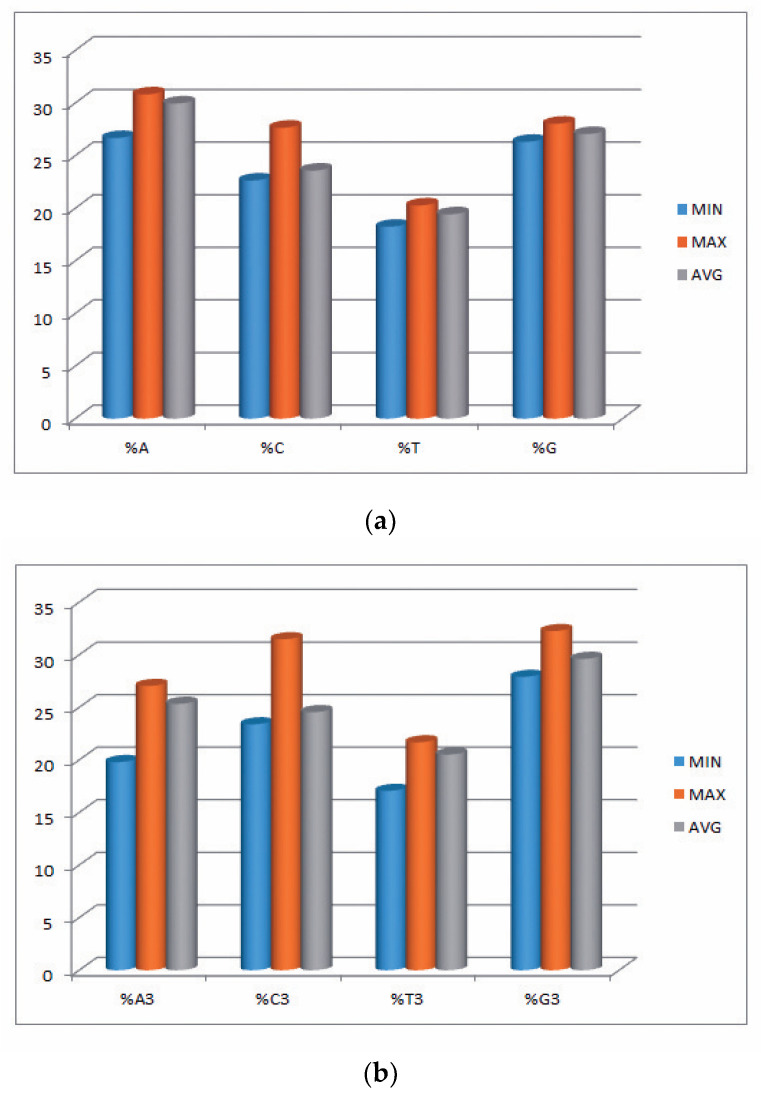
(**a**) Minimum, maximum, and average values of nucleobases A, C, T, and G of the EPB41L3 gene. (**b**) Minimum, maximum, and average of nucleobases at third position %A3, %C3, %T3, and %G3 of the EPB41L3 gene.

In the current study, we explored 34 transcripts comprising 29,660 codons and 88,980 nucleotides of the EPB41L3 gene across five different mammalian species. Insights into the compositional properties of the transcripts disclosed that the overall A% (29.95 ± 0.95%) was preeminent, followed by G% (27.05 ± 0.40%) (Table S2). The nucleotide contents, particularly at the third codon position (A3, U3, G3, and C3), showed the overall G3% (29.65 ± 1.11%) was the highest, followed by A3% (25.45 ± 1.47%). This result supported that there might be more usage of the nucleobases A and G (purines) over the nucleobases C and U (pyrimidines) among the codons of the EPB41L3 gene. However, the mean GC% of 50.62 ± 1.23% (Table 2) and AU% of 49.37 ± 1.23% (Table S2) revealed almost equal GC% and AU% content. Further nucleotide composition analysis indicated that the mean GC3% and AU3% compositions were 53.95 ± 2.27% (ranging between 51.60–63.10) and 46.05 ± 2.28% (ranging between 36.86–48.37), respectively (Table S2). Consequently, insight into the overall nucleotide content usage supported the G/C-ending codons preference over the codons ending with A/U in the EPB41L3 gene among the envisaged species. The minimum, maximum, and mean values of nucleobases along with the nucleobases at the third position of the EPB41L3 gene are presented graphically in Figure 1a,b.

### 3.2. Relative Dinucleotide Abundance Analysis Indicated GpA as the Most Abundant Dinucleotide Owing to Overall High GA Nucleotide Content

**Figure 2:**
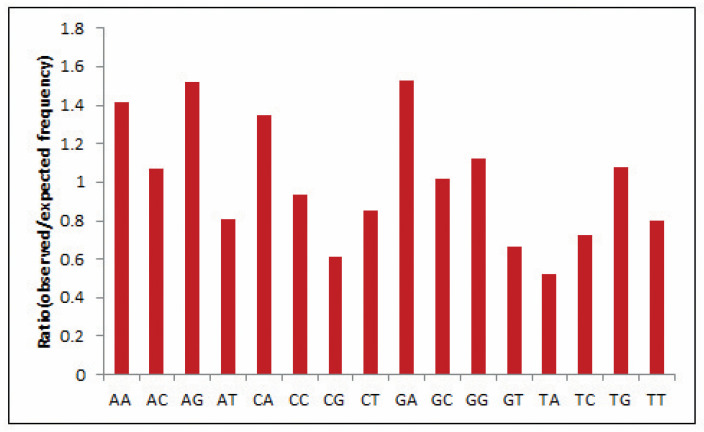
Relative dinucleotide frequencies among the EPB41L3 transcripts.

Dinucleotide composition is an efficient tool to predict bias as the compositions typically vary across species and are strongly symmetrical within a single genome [31]. Hence, often, the genome’s odds ratio profile (as discussed above) pertains to its genomic characteristics [32]. In the present study, the most abundant dinucleotide was found to be GpA with an odds ratio of 1.524, reflecting high GA content in the EPB41L3 gene, whereas dinucleotide pair UpA with an odds ratio of 0.522 was the lowest and underrepresented (less than 0.78) (Table 3). Relative dinucleotide frequencies among the EPB41L3 transcripts are shown in Figure 2.

### 3.3. Relative Synonymous Codon Usage in the EPB41L3 Gene Revealed Preference of GpA-Ending Codons across the Selected Mammalian Species

**Figure 3:**
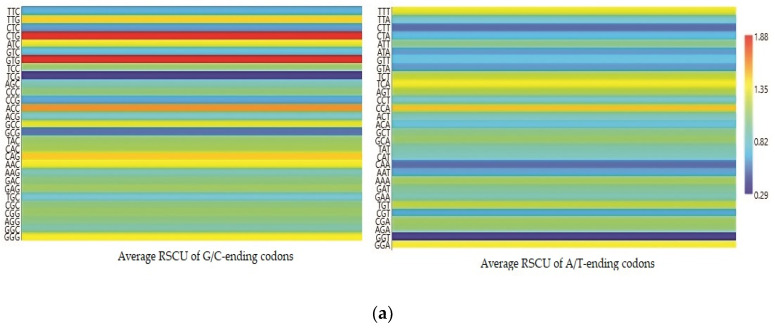

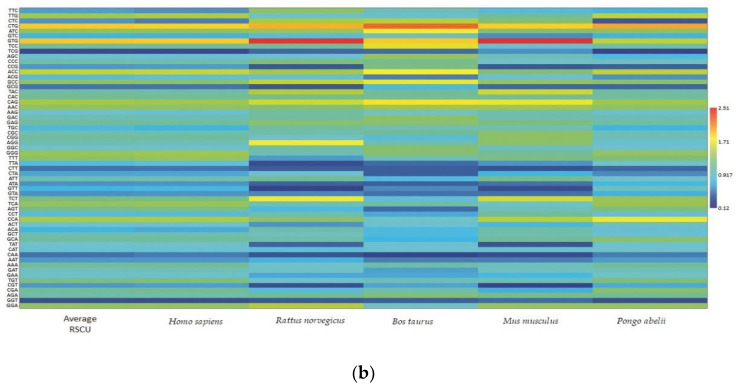
(**a**) Matrix plot of the average RSCU values against the distribution of G/C-ending and A/T-ending codons. The overrepresented (RSCU > 1.6), underrepresented (RSCU < 0.6), more frequently (RSCU > 1), and less frequently used codons (RSCU < 1) are shown. (**b**) Clustering of RSCU values of the EPB41L3 gene across the selected mammals. Heat map comparing the average RSCU value of a codon (rows) corresponding to the gene transcripts across the mammalian species (columns). The map indicates differing codon preferences within the gene itself (higher RSCU values with more frequent codon usage depicted in dark red and lower RSCU values depicted in dark blue).

Each codon’s RSCU value was computed to interpret how recurrently G/C-ending codons could be favored (Table 1). The average RSCU values of all the synonymous codons corresponding to 18 amino acids of the EPB41L3 gene along with the selected individual mammalian species were analyzed. The overall RSCU value analysis of the EPB41L3 gene suggested that 31 codons were frequently used (RSCU > 1), where G/C-ending codons (18) were predominantly used in contrast to A/U-ending codons (13) (Table 1). Additionally, C-ending codons (10) were preferred over G-ending codons (8) in the EPB41L3 transcripts across the envisaged mammalian species. The findings also indicate the GC content preference as usually preferred among the eukaryotic genomes in the EPB41L3 gene amongst all the five envisaged mammalian species. Among the 31 most frequently used codons, the present study revealed that all the UpA-ending codons were not preferred (RSCU value below 1), whereas all the GpA-ending codons were preferred (RSCU value above 1) across the selected mammalian species. Moreover, among the most frequently used codons, 14 codons, CUG (leucine), AUC (isoleucine), GUG (valine), ACC (threonine), GCC (alanine), UAC (tyrosine), CAC (histidine), CAG (glutamine), AAC (asparagine), GAC (aspartic acid), GAG (glutamic acid), CGC, AGA (arginine), and GGG (glycine), were found common across the selected mammalian species, depicting the evidence of a shared codon preference. More insight into RSCU values (Table 1) showed that the codons CUG, GUG (except for *Pongo abelii*), CAG (except for *Homo sapiens* and *Pongo abelii*), and UCU (except for *Homo sapiens*, *Bos taurus*, and *Pongo abelii)* were overrepresented while some codons such as AUC, UCC, ACC, and GCC (*Bos taurus*), CCA (*Pongo abelii)*, UAC (*Mus musculus*), and AGA (*Rattus norvegicus*) were found overrepresented for individual species. The matrix plot (Figure 3a) drawn using the average RSCU values of codons showed a remarkable difference in codon usage in the EPB41L3 gene. Furthermore, the heat map represented the same inference that G/C-ending codons were favored over A/U-ending codons among the synonymous codons of the EPB41L3 gene (Figure 3b). The maximum overall RSCU value was observed for the codon GUG (1.88; valine) preceded by CUG (1.86; leucine) across the selected mammalian species. The RSCU values >1 (more frequently used codons) are highlighted in bold and asterisk-marked, the shared preferred synonymous codons across the envisaged mammalian species are highlighted in bold, and the overrepresented codons are highlighted in red (Table 1).

### 3.4. Neutrality Plot Showed Dominance of Selection Pressure

**Figure 4:**
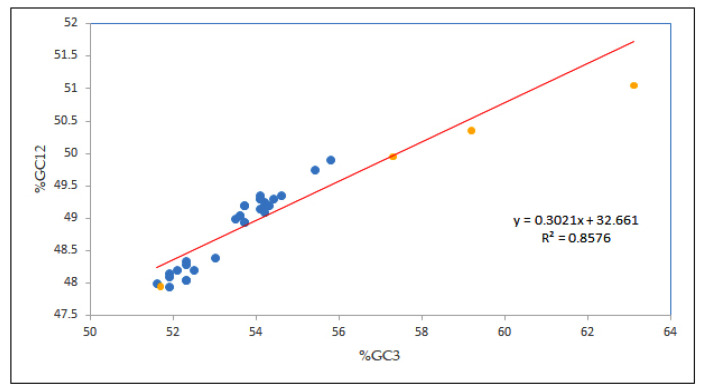
Neutrality plot analysis (GC12 vs. GC3) for the entire coding sequences of EPB41L3. GC12 stands for the average value of GC contents at the first and second positions of the codons (GC1 and GC2), while GC3 refers to the GC contents at the third codon position. The red line is the linear regression of GC12 against GC3, R = 0.926, relative neutrality is 30.21%. The blue dots are assigned for *Homo sapiens* while the yellow dots are assigned for other mammals.

In this study, to figure out the strength of mutational and selection forces in determining the CUB of the EPB41L3 gene, neutrality analysis was performed [29]. A change at the third codon position results in a synonymous codon, that is, a corresponding amino acid is not changed, and thus there is no contribution of the selection force [15]. The strong positive correlation coefficient of GC12 and GC3 indices implies the influence of the mutational force on the codon utilization pattern [33]. Our result showed a notable high positive correlation (r = 0.926, *p* < 0.00001; GC12 versus GC3) inferring the mutational forces acting throughout the codon positions. Moreover, the regression line slope <0.5 indicates influence of the selection pressure [24]; in the present study, the regression slope of was 0.302 (Figure 4), representing the neutrality of 30.21% vis-à-vis, the mutational force was 30.21%, and the selection force was 69.79%, demonstrating the role of natural selection in shaping the codon usage of the EPB41L3 gene.

### 3.5. Parity Analysis Indicated Predilection for A/G over U/C at the Third Codon Position Owing to Selection Pressure

According to Chargaff’s PR2 rule, in a DNA strand, the residue of A is equal to T and the residue C is equal to G [34], and at the coordinate position (0.5, 0.5) of the plot, no biasness between the mutation and selection rates has been reported [24].

**Figure 5:**
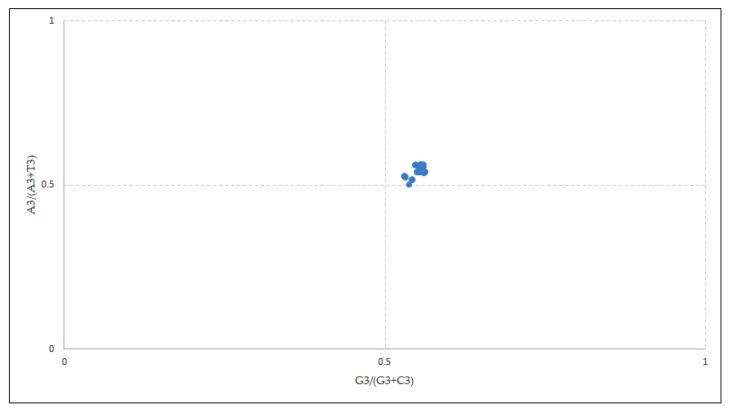
Parity analysis showing preference of purine over pyrimidine. A disproportionate distribution shows the preference towards codon choices is shaped by both mutational pressure and natural selection.

The overall AT bias [A3/(A3 + T3)] was 0.552 and the GC bias [G3/(G3 + C3)] was 0.549 (Figure 5). As per the parity plot, the values were not situated in the center. An unequal distribution might refer to the involvement of both mutational and selection forces in deciding the biasness [35]. In the EPB41L3 gene, purines were preferred over pyrimidines as the PR2 > 0.5 points towards the predilection for A/G over U/C at the third codon position [36] that confirms the selection pressure.

### 3.6. Codon Adaptation Index Close to 1 Shows Better Adaptation

Furthermore, the codon utilization preferences of the EPB41L3 gene among the selected mammalian species were examined using the CAI. The CAI determines the degree of the translation selection force acting upon a gene and thus plays a role in the directional measure of the CUB [19]. The EPB41L3 transcripts’ computed CAI values using the codon usage database (https://www.kazusa.or.jp/codon/, accessed on 2 October 2020) of the respective mammalian species are provided in Table S2. The mean CAI value for all the 34 CDSs of the EPB41L3 gene was found to be 0.77 ± 0.01 (Table 2). CAI values range between 0 and 1. Sequences having the CAI values approaching closer to 1 are found to be better suited for a certain host than those with the CAI closer to 0 [15]. The correlation analysis between various CUB indices is provided in Table 4a.

### 3.7. Mutational Force Plays a Minor Role in Configuring the CUB of the EPB41L3 Gene

**Figure 6:**
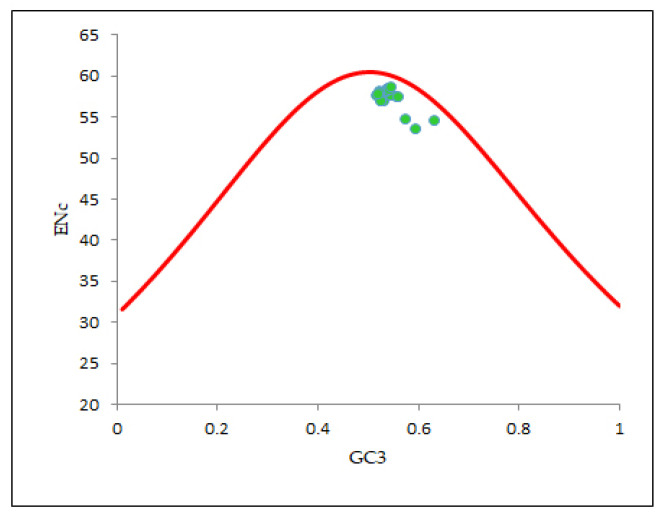
ENc-GC3 plot (selection curve) analysis. ENc denotes the effective number of codons and GC3s denotes the GC content in the third synonymous codon position. The red line curve represents the expected curve when the codon usage was only determined by the GC3 composition.

For determining the intent of mutation pressure in shaping the codon utilization trends in the EPB41L3 gene, an ENc-GC3 plot was constructed. Wright (1990) [20] recommended that resulting points collapse exactly onto the anticipated curve in the plot if GC3s are the only driving SCU patterns. In our ENc-GC3 plot (Figure 6), all the relevant points were found substantially below the anticipated curve, indicating that mutation is not the prime factor, unlike other evolutionary factors including selection forces that tend to be associated with regulation of the specific restraints in configuring the CUB of the EPB41L3 gene [29,37]. A noteworthy negative correlation (r = −0.7131, *p* < 0.001) was found between ENc-GC3s (Table 4b).

### 3.8. Relevance of Bias in the Use of Codons and Compositional Attributes

**Figure 7:**
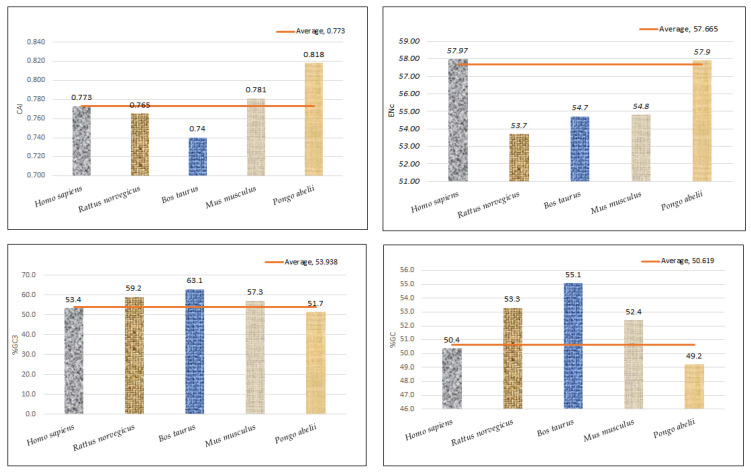
Graphical representation of CAI, ENc, GC3%, and GC% with their mean values.

**Figure 8:**
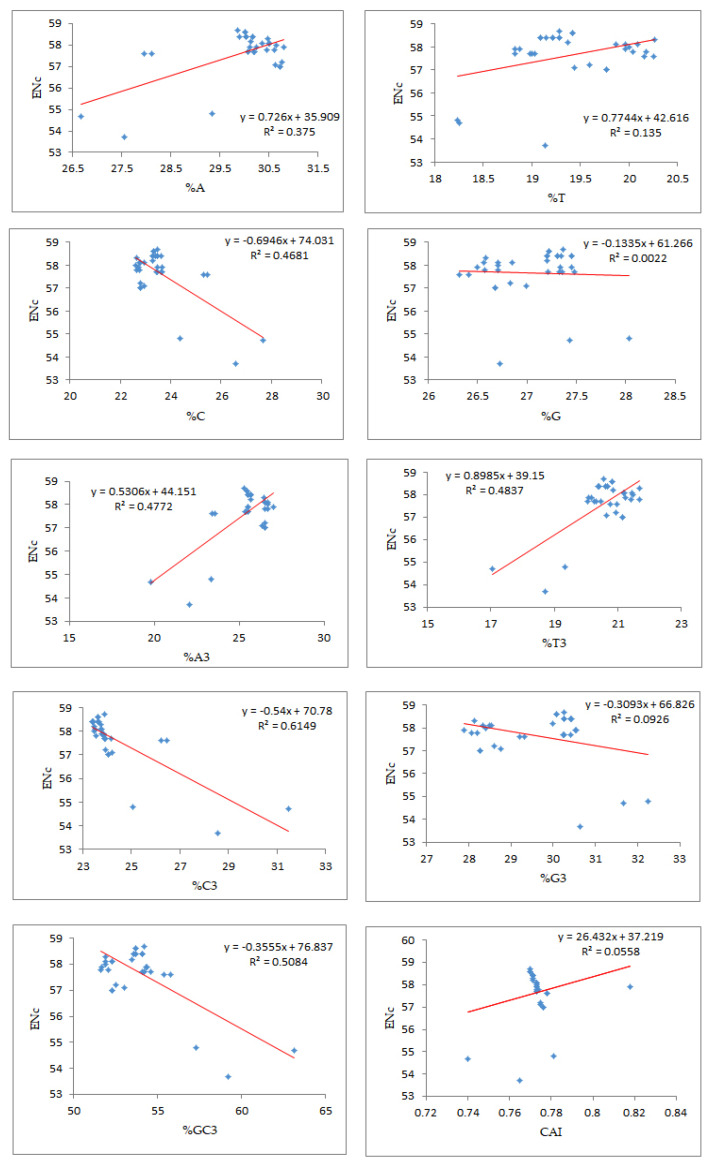
Regression analysis of the ratio of the ENc and the compositional properties, %GC3, and CAI.

Regression analysis between the mean ENc and compositional attributes was performed to analyze the effects of selection pressure on CUB patterning of the EPB41L3 gene. The ENc is a nondirectional measure of the CUB and depends upon the composition of a gene [38]. The higher the ENc, the lower the CUB, whereas a gene with the ENc value inclining towards the lower range than the gene consists of optimal codons and can be associated with elevated translational efficiency [39]. The mean ENc for the EPB41L3 transcripts is 57.66 ± 1.132 (Table 2), inferring low CUB (ENc > 35). The ENc values of the selected mammalian species with an average value of 57.66 (Figure 7) depicted a nearly similar (very low) CUB. The regression plot was drawn between the ENc and different CUB indices (Figure 8). The regression coefficient was positive for A, T, A3, T3, and CAI and negative for C, G, C3, G3, and GC3. The negative values of the regression coefficient of the ENc with C, G, C3, G3, and GC3 infer a positive influence on the CUB.

### 3.9. P2 Analysis Indicated High Expression Level of the EPB41L3 Gene among the Envisaged Species and Dominance of Translational Selection

The values of SSU, WWU, SSC, and WWC were computed using the RSCU values of their corresponding codons (Table 5). The overall P2 value of the EPB41L3 gene was 0.97 (Table 2), implying higher translational efficacy of the respective gene. The EPB41L3 gene amongst the species showed P2 > 0.5 (Table 5), indicating that translational selection has the dominant role over the mutational force in the codons’ utilization patterns.

### 3.10. Codon Utilization Trends in the EPB41L3 Gene Harmonize to the Phylogeny of the Selected Species and the Homo sapiens’s EPB41L3 Gene Resembles the Pongo abelii’s EPB41L3 Gene

**Figure 9:**
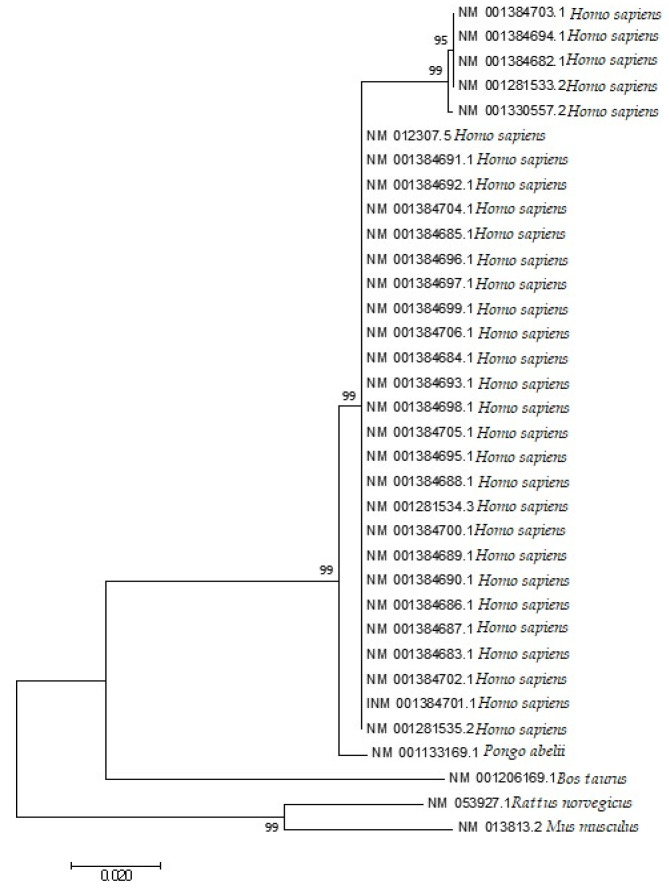
Phylogenetic analysis based on the Kimura two-parameter (K2P) distances of the mRNA coding sequences of the EPB41L3 gene of different mammalian species. The evolutionary analyses were performed in MEGA7, whereas the evolutionary distances were calculated using the Kimura two-parameter method and are presented in the number of base substitutions per site.

The phylogenetic analysis (Figure 9), a neighbor-joining method under the principle of minimum evolution [40], following K2P distances of the EPB41L3 transcripts covering the five selected mammalian species was performed. The neighbor-joining tree analysis showed that the codon utilization trends in the EPB41L3 transcripts have notable resemblance among the intently connected mammalian species. The gene EPB41L3 in *Rattus norvegicus* indicated similarities to the EPB41L3 gene in *Mus musculus*; likewise, this gene in *Homo sapiens* resembled that of *Pongo abelii*. Moreover, in a study, it was inferred that genes with similar functions have a decisive role in shaping similar patterns of codon utilization while species play a supportive character in deciding the further difference in the CUB for genes with similar functions [41].

### 3.11. Abundance of tRNA Influences Gene Expression and the Indicated Codon Preference Does Not Correspond to the Most Abundant tRNA Pool

The tRNA pool shown (Table 6a–e) represents the frequencies of tRNA genes in human cells along with four other mammalian cells. Our study indicated that in the EPB41L3 gene, the most preferentially and commonly shared codons across all the five mammalian species were for the amino acids Val, Asn, Asp, His, Gln, and Tyr. Their respective codons GTG, AAC, GAC, CAC, CAG, and TAC were preferred at these six codon–anticodon positions. These six codon–anticodon positions (Val, Asn, Asp, His, Gln, and Tyr) correspond to the most abundant tRNA isotypes present in human cells. *Rattus norvegicus* and *Mus musculus* showed marked similarities in their preferred codon families and, likewise, *Homo sapiens* and *Pongo abelii* exhibited similarities in their preferred codon families. Moreover, on comparing the synonymous optimal codons with their respective tRNA anticodon of each of the envisaged five mammalian species individually, amino acids, namely Leu and Glu in *Homo sapiens*, Arg, Leu, Phe, Lys, and Cys in *Rattus norvegicus*, Gly, Arg, Phe, Lys, and Cys in *Bos taurus*, Pro, Ser, Arg, Leu, Lys, Glu, and Cys in *Mus musculus*, and Ser, Lys, and Cys in *Pongo abelii* were found to have optimal codon–anticodon usage (here, the most preferred codon had highly abundant tRNA isotypes) except for Trp and Met. Overall, these outcomes supported much less adaptation between the codon usage preference for EPB41L3 and the tRNA pool corresponding to the envisaged mammalian species cells.

## 4. Discussion

The present study envisaged synonymous codon usage in tumor suppressor protein-coding gene EPB41L3 among the five mammalian species that were identified. Nucleotide composition is an imperative factor in determining codon utilization in genes and genomes [42]. GC3 content at the third codon position signifies codon usage bias as Shen et al. (2015) [26] suggested that genes with a significant amount of GC3 content co-translate in specific spatial regions and might be involved in genome organization. Genes with a higher GC content present a greater number of targets for methylation [43] as the degree of methylation plays a significant role in alteration of the gene expression level [44]. In previous studies, hypermethylation of tumor suppressor genes has been found linked with brain tumor progression. Furthermore, the EPB41L3 gene has been found hypermethylated (29% methylation) only in tumors, confirming its cancer-specific role [7]. In another study, inactivation of the EPB41L3 gene through methylation was involved in breast cancer and renal clear cell carcinoma [1]. Along with this, in the development and progression of NSCLC, hypermethylation also plays an imperative role and exacerbates a poor prognosis [6]. The overall GC content (53.8%) of the AARS (alanyl-tRNA synthetase) gene that belongs to the family of tRNA synthases of class II enzymes helps in tRNA aminoacylation and gene expression was found higher than the AT (46.2%) content [45,46]. GATA2 has a key role in hematopoietic development and the key to KRAS-driven non-small-cell lung cancer; its GC content (65.2%) is higher than the AT content (34.8%) [47,48]. In the present study, the average GC content was 50.63 ± 1.24% in the EPB41L3 gene among the selected mammalian species that was close to half of the total nucleotide content, indicating that a critical balance of methylation was over to proper functioning of the EPB41L3 gene.

The set of dinucleotide odds ratios is specific to every genome and often referred to as a signature. Closely related organisms often exhibit similar odds ratios in comparison to those which are distantly related [49], and thus they can help discriminate within and between species. Species-specific properties, including DNA modification, replication, and repair mechanisms, are reflected by the dinucleotide odds ratio [50]. In this study, relative dinucleotide abundance analysis (Table 3) revealed that the most abundant dinucleotide was GpA with an odds ratio of 1.524, reflecting high GA dinucleotide content in the EPB41L3 gene, whereas dinucleotide pair UpA with an odds ratio of 0.522 was the lowest and underrepresented (less than 0.78). In previous studies, mutations in breast cancers (31%) and in colorectal cancers (11%) were found at 5′-GpA-3′ sites (or at complementary 5′-TpC-3′ sites). UA (TA) is an obligatory component of various regulatory sequences of both prokaryotic and eukaryotic origin. The examples include TATA box in prokaryotes, TATATA in yeast, and polyadenylation signals, i.e., AATAAA in higher eukaryotes; therefore, this dinucleotide is used in a restrictive manner to avoid inappropriate binding of regulatory elements [51], and the EPB41L3 gene is not the exception.

A remarkable difference in codon usage in the EPB41L3 gene was observed through the matrix plot (Figure 3a) using the average RSCU values of codons. The G/C-ending codons were majorly preferred amongst the frequently used codons having the RSCU > 1. The most preferable codons (RSCU > 1.6) ended with G/C in all the degenerate codons across the selected mammalian species (except UCU for serine) (Table 1). Among the 31 most frequently used codons, 18 ended with G/C, and two codons, namely GUG and CUG that code for valine and leucine with the highest average RSCU values of 1.88 and 1.86, correspondingly, were considered in the affirmation of a shared codon preference. Relative dinucleotide abundance analysis supported the preferred usage of GpA-ending codons over UpA-ending codons across the selected mammalian species.

According to Wright (1990) [20], the ENc determines the effect of the selection force on the codon utilization pattern of any gene. A higher ENc value exhibits a lower bias in codon usage. Low codon usage bias, as its name itself indicates, shows an almost equal use of the synonymous codons for their corresponding amino acids in CDSs [5]. Low CUB presents an advantage for organisms growing on other organisms as well as cells with different codon choices [33]. The average ENc value for the EPB41L3 gene was found to be 57.66 ± 1.132, which suggested that the CUB was low. A similarity in the results was found for the study involving genes of the Coronaviridae family, where the ENc close to 50 suggested a low mutational force in shaping the CUB [52]. The %GC3 value ranged between 51.60 and 63.10 with an overall value of 53.95 ± 2.27 (Table S2). A noteworthy negative correlation (r = −0.7131, *p* < 0.001) was found between ENc-GC3s (Table 4b). The regression plot of the ENc versus GC3 (Figure 8) had a negative regression coefficient that inferred a positive influence on the CUB [53]. In addition, the selection curve, a plot between the ENc and GC3s (Figure 6) is suggestive of non-exclusiveness of the mutational force in shaping codon usage and also indicates the presence of other factors including natural selection associated with shaping codon usage in the EPB41L3 gene [29,37,45].

Different computational parameters were used to illustrate the role of mutational and/or selection pressure on codon utilization of the EPB41L3 gene. In this study, in Figure 8, we found significant negative regression coefficients of the ENc against nucleobases (C, G, G3, C3, and GC3) indicating a positive influence on codon usage bias. An earlier study on genes implicated in the CNS showed similar evidence of negative regression coefficients of the ENc against nucleobases G, C, G3, and C3 [54].

Neutrality plot analysis depicts a relation of GC12 versus GC3; it is performed to figure out the effect of mutational and/or selection pressure in configuring the codon utilization patterns [29]. A mutation may occur without any known external pressure. If a mutation occurs at the third codon position, it culminates with synonymous substitution, i.e., the corresponding amino acid is not changed and thus there is no contribution of selection, whilst mutations at the first or second position of the codon result in non-synonymous changes leading to changes in the amino acid [15]. In the present study, a notable positive correlation between GC12 and GC3 (r = 0.926 *p* < 0.00001) was found. A similarity in the results was found for the study involving structural and non-structural genes of the Coronaviridae family, where all the genes (E, M, N, S, ORF1a, and ORF8) had positive correlations between GC12 and GC3; all the structural genes except the S gene (correlation value above 0.6 with *p* < 0.05) had a greater influence of selection pressure over mutational forces on the CUB [52].

A study encompassing the yeast *URA3* gene with a variable GC content (31%, 43%, and 63%) revealed that in the gene containing high GC%, the mutation rate was elevated with the presence of both single-base substitutions and deletions [55] attributed to DNA polymerase slippage. The GC-rich genes also exhibit higher rates of mitotic and meiotic recombination indicating an important role of the GC content in genome evolution. The average GC3 content is high in the EPB41L3 gene; hence, it is speculated that the gene is more prone to mutation. High GC content is supposed to facilitate more complex gene regulation [56]. Since CpG dinucleotides are prone to methylation and the degree of methylation alters the gene expression level [44] and ultimately affects phenotypes [44] apart from other genomic factors, these affect gene expression levels and result in new phenotypes [57]. The GC- and AT-rich domains in a gene display distinct chromatin conformations and histone modifications. The transcription process of coding sequences gets slowed down due to high GC content in the transcription bubble [58]. The GC content and gene expression are highly correlated [59]; however, the same is debated in mammals. To further assess the effect of the GC content on methylation, translational selection was calculated. The average P2 value of the EPB41L3 gene was 0.97, indicating high translational efficiency, inferring that methylation probably has no effect on gene expression.

According to the “genome hypothesis” [60], the codon preference patterns are considered to be well-conserved during the course of evolution. The pattern of synonymous codon usage is different between different kinds of organisms. The choice of synonymous codons is similar in all genes for a particular genome. Furthermore, within an organism, codon choice is linked to organism-specific isoaccepting tRNAs [61,62]. We compared the most preferred codon families of the EPB41L3 gene related to the selected mammalian tRNAs pool. According to the tRNA frequency tables (Table 6), the most preferentially and commonly shared codons across all the five mammalian species were for the amino acids Val, Asn, Asp, His, Gln, and Tyr. Their respective codons GTG, AAC, GAC, CAC, CAG, and TAC were preferred at these six codon–anticodon positions. Cluster analysis (neighbor-joining method) was performed based on the K2P distances of the CDSs in the EPB41L3 gene across the five selected mammalian species (Figure 9); the result showed a similar resemblance to the CUB pattern in the EPB41L3 gene across the envisaged five mammalian species. A study suggested that genes with similar functions have a decisive role in shaping similar patterns of codon utilization, while species play a supportive character in deciding the further difference in the CUB for genes with similar functions [12]. Moreover, when comparing the synonymous optimal codons with their respective tRNA anticodon of each of the envisaged five mammalian species individually, amino acids, namely Leu for *Homo sapiens*, *Rattus norvegicus*, and *Mus musculus*, Cys and Lys for *Rattus norvegicus*, *Bos taurus*, *Mus musculus*, and *Pongo abelii*, and Ser for *Rattus norvegicus*, *Mus musculus*, and *Pongo abelii* showed similar codon preferences and were found to have optimal codon–anticodon usage (here, the most preferred codon had highly abundant tRNA isotypes) except for Trp and Met. The impact of the tRNA choice has been found to affect the evolution of different codon choice patterns in early/late genes in viruses [63].

Overall, these outcomes supported much less adaptation between the codon usage preference for EPB41L3 and the tRNA pool corresponding to the envisaged mammalian species cells. However, a previous study conducted in the human genome by Comeron [64] observed that in a highly expressed gene, a combination of the most preferred codon and its respective most abundant tRNA gene was present.

## 5. Conclusions

In this study, we investigated the compositional properties and biases in the codon utilization trends of the gene were envisaged as the quantity of protein expressed from the coding sequences may vary remarkably due to distinguishable translational properties of different synonymous codons under evolutionary forces. In brief, our results indicated a fairly low CUB within the gene due to the high ENc value. Out of 31 frequently used codons, 18 ended with G/C and two overrepresented codons (GUG and CUG) were identified across all the selected mammalian species. Dinucleotide odds ratio values reflected the high quantity of GA in the EPB41L3 gene, whereas the dinucleotide pair UpA was very low. Codon usage in the EPB41L3 transcripts among the envisaged species was significantly affected by the GC bias, primarily due to GC3. P2 value (*>*0.5) indicated high translational efficiency of the EPB41L3 gene that implies the presence of optimal codons, inferring that methylation has probably no effect on gene expression. A disproportionate distribution in the parity plot might refer to the involvement of both mutational and selection forces in deciding the biasness; moreover, PR2 > 0.5 indicated the preference of purine over pyrimidine at the third codon position that confirms the influence of selection pressure in the EPB41L3 transcripts. Additionally, neutrality plot, ENc-GC3, and parity analysis inferred the dominance of selection pressure over mutational pressure throughout the codon positions, suggesting that natural selection tends to be associated with regulating the specific restraints on patterning the codon usage in the EPB41L3 gene. The negative values of the regression coefficient of the ENc with C, G, C3, G3, and GC3 infer a positive influence on the CUB. The phylogeny analysis and heat map of the RSCU values showed resemblance in the codon usage pattern of EPB41L3 in *Homo sapiens* to that of *Pongo abelii*, as well as in *Rattus norvegicus* to that of *Mus musculus*. Our study revealed that a specific gene with similar functions in closely related species shows a similar trend in codon usage as perceived from an earlier study in the serotonin receptor gene family [39]. Investigation of the preferred codons used by EPB41L3 and the corresponding tRNA pools of the envisaged species inferred that the EPB41L3 transcripts do not prefer codons from their corresponding suboptimal anticodon tRNA pool. The overall codon usage pattern study disclosed that the selection is the key factor influencing the pattern of codon utilization in the EPB41L3 gene.

## Figures and Tables

**Table 1:** Relative synonymous codon usage (RSCU) analysis of the various codons of all the 34 coding sequences of the EPB41L3 gene of the selected mammalian species.

S. No.	Amino Acid	Codons	RSCU EPB41L3					
			*Homo sapiens*	*Rattus norvegicus*	*Bos taurus*	*Mus musculus*	*Pongo abelii*	Average of the Species
1	Phenylalanine (F)	**UUU**	1.36 *	0.67	1.0	1.2 *	1.25 *	1.32 *
		UUC	0.64	1.33 *	1.0	0.8	0.75	0.68
2	Leucine (L)	UUA	0.83	0.32	0.53	0.65	0.93	0.80
		UUG	1.49 *	0.9	0.86	1.11 *	1.55 *	1.44 *
		CUU	0.56	0.45	0.46	0.37	0.52	0.55
		CUC	0.59	1.48 *	1.52 *	1.29 *	0.41	0.65
		CUA	0.70	0.9	0.46	0.74	0.62	0.70
		CUG	1.84 *	1.94 *	2.18 *	1.85 *	1.97 *	1.86 *
3	Isoleucine (I)	AUU	1.03 *	1.14 *	0.79	1.18 *	0.92	1.02 *
		AUC	1.32 *	1.38 *	1.74 *	1.26 *	1.31 *	1.33 *
		AUA	0.65	0.49	0.47	0.55	0.77	0.64
4	Valine (V)	GUU	0.77	0.14	0.62	0.31	0.98	0.74
		GUC	0.73	0.81	0.97	0.63	0.73	0.74
		GUA	0.65	0.61	0.55	0.55	0.73	0.64
		GUG	1.86 *	2.44 *	1.86 *	2.51 *	1.55 *	1.88 *
5	Serine (S)	UCU	1.18 *	1.72 *	0.83	1.62 *	1.39 *	1.21 *
		UCC	1.08 *	1.1 *	1.66 *	0.85	1.04 *	1.09 *
		UCA	1.39 *	0.99	1.07 *	0.92	1.39 *	1.36 *
		UCG	0.27	0.52	0.53	0.64	0.17	0.29
		AGU	1.19 *	0.78	0.53	0.99	1.22 *	1.16 *
		AGC	0.87	0.89	1.37 *	0.99	0.78	0.89
6	Proline (P)	CCU	0.80	1.01 *	0.71	1.11 *	0.85	0.81
		CCC	1.05 *	1.25 *	1.15 *	0.94	0.97	1.05 *
		CCA	1.49 *	1.31 *	0.93	1.53 *	1.7 *	1.47 *
		CCG	0.67	0.42	1.21 *	0.43	0.48	0.66
7	Threonine (T)	ACU	0.89	0.65	0.88	0.77	0.96	0.88
		ACC	1.58 *	1.45 *	1.76 *	1.28 *	1.55 *	1.57 *
		ACA	0.71	1.05 *	0.78	1.08 *	0.85	0.74
		ACG	0.83	0.85	0.59	0.87	0.64	0.82
8	Alanine (A)	GCU	1.03 *	0.97	0.78	1.01 *	0.87	1.02 *
		GCC	1.31 *	1.56 *	1.7 *	1.35 *	1.31 *	1.33 *
		GCA	1.10 *	1.06 *	0.74	1.13 *	1.31 *	1.09 *
		GCG	0.56	0.41	0.78	0.51	0.51	0.56
9	Tyrosine (Y)	UAU	0.94	0.5	0.88	0.4	0.9	0.91
		UAC	1.06 *	1.5 *	1.12 *	1.6 *	1.1 *	1.09 *
10	Histidine (H)	CAU	0.87	0.84	0.89	0.89	0.92	0.87
		CAC	1.13 *	1.16 *	1.11 *	1.11 *	1.08 *	1.13 *
11	Glutamine (Q)	CAA	0.58	0.4	0.2	0.31	0.58	0.55
		CAG	1.42 *	1.6 *	1.8 *	1.69 *	1.42 *	1.45 *
12	Aspargine (N)	AAU	0.66	0.73	0.59	0.59	0.67	0.66
		AAC	1.34 *	1.27 *	1.41 *	1.41 *	1.33 *	1.34 *
13	Lysine (K)	AAA	1.14 *	0.92	1.07 *	0.97	1.13 *	1.13 *
		AAG	0.86	1.08 *	0.93	1.03 *	0.87	0.88
14	Aspartic acid (D)	GAU	0.98	0.92	0.68	0.96	0.98	0.97
		GAC	1.02 *	1.08 *	1.32 *	1.04 *	1.02 *	1.03 *
15	Glutamic acid (E)	GAA	0.90	0.7	0.7	0.77	0.93	0.89
		GAG	1.10 *	1.3 *	1.3 *	1.23 *	1.07 *	1.11 *
16	Cysteine (C)	UGU	1.25 *	0.92	1.0	1.0	1.25 *	1.22 *
		UGC	0.75	1.08 *	1.0	1.0	0.75	0.78
17	Arginine (R)	CGU	0.68	0.35	0.69	0.12	0.71	0.66
		CGC	1.03 *	1.04 *	1.15 *	1.32 *	1.0	1.04 *
		CGA	1.13 *	0.81	1.04 *	0.72	1.29 *	1.11 *
		CGG	1.10 *	0.92	0.81	1.32 *	1.0	1.09 *
		AGA	1.06 *	1.15 *	1.27 *	1.2 *	1.14 *	1.07 *
		AGG	0.99	1.73 *	1.04 *	1.32 *	0.86	1.02 *
18	Glycine (G)	GGU	0.34	0.43	0.47	0.53	0.34	0.35
		GGC	0.89	1.07 *	1.29 *	0.98	0.94	0.91
		GGA	1.36 *	1.5 *	0.95	1.36 *	1.36 *	1.35 *
		GGG	1.41 *	1.0	1.29 *	1.13 *	1.36 *	1.39 *

The values with more frequently used codons (RSCU > 1) corresponding to each amino acid of the gene transcripts among each species are highlighted in bold and asterisk-marked and the values highlighted in red were found overrepresented (RSCU > 1.6). Bold codons showed shared preferred synonymous codons across the envisaged mammalian species.

**Table 2:** Total codons count for all the 34 transcripts of the EPB41L3 gene with the average values of CAI, GC%, GC1%, GC2%, GC3%, ENc, and P2.

Gene	No. of CDSs Evaluated	Total Number of Codons	Average CAI Value ± SD	Average %GC ± SD	%GC1 ± SD	%GC2 ± SD	%GC3 ± SD	ENc ± SD	P2 ± SD
EPB41L3	34	29,660	0.773 ± 0.010	50.626 ± 1.231	55.773 ± 0.970	42.144 ± 0.626	53.953 ± 2.271	57.656 ± 1.132	0.97 ± 0.03

**Table 3:** The occurrence of the dinucleotide odds ratio in the EPB41L3 gene. The odds ratio was calculated by dividing each observed frequency by the expected frequency of dinucleotides.

S. No.	Dinucleotide	Observed Frequency	Expected Frequency	Odds Ratio
1	ApA	0.0883375	0.0625	1.4133997
2	ApC	0.0669078	0.0625	1.0705248
3	ApG	0.0948584	0.0625	1.5177349
4	ApU	0.0504045	0.0625	0.8064717
5	CpA	0.0842667	0.0625	1.348268
6	CpC	0.0584552	0.0625	0.9352831
7	CpG	0.0382312	0.0625	0.6116988
8	CpU	0.053101	0.0625	0.8496163
9	GpA	0.0952992	0.0625	1.5247874
10	GpC	0.0635631	0.0625	1.0170089
11	GpG	0.0701099	0.0625	1.121759
12	GpU	0.0415111	0.0625	0.6641776
13	UpA	0.0326048	0.0625	0.521676
14	UpC	0.0451281	0.0625	0.7220494
15	UpG	0.0672838	0.0625	1.0765401
16	UpU	0.0499378	0.0625	0.7990044

The odds ratio values highlighted with green and red fonts correspond to the maximum and minimum values for the GpA and UpA dinucleotides, respectively.

**Table 4:** (**a**) Correlation analysis between GC3%, ENc, CAI, and length of transcripts. (**b**). Correlation between the ENc and compositional properties of the EPB41L3 gene transcripts was performed to de-termine the effect of nucleotide bases on the CUB. The positive correlation between the ENc and A, A3, and T3 and negative correlation between C, C3, and GC3 inferred the role of both natural selection and mutation pressure on the CUB in the EPB41L3 gene.

(**a**)											
	**Y**	**GC3%**		**ENc**		**Length of Transcripts**					
**X**											
ENc		−0.3042 ***		-		−0.425 **					
CAI		−0.542 **		0.2362		−0.3266					
(**b**)											
		**A%**	**T%**	**G%**	**C%**	**A3%**	**T3%**	**G3%**	**C3%**	**GC3%**	**CAI**
ENc		0.6124 ***	0.3675 **	−0.0471	−0.6842 ***	0.6908 ***	0.6955 ***	−0.3042	−0.7841 ***	−0.7131 ***	0.2362

Here, *** *p* < 0.001; ** *p* < 0.01.

**Table 5:** The values of SSU, WWU, SSC, WWC, and P2 of the EPB41L3 gene in five species.

Species	SSU	WWU	SSC	WWC	P2
*Homo sapiens*	0.71	1.00	1.07	1.09	0.93
*Rattus norvegicus*	0.69	0.76	1.23	1.37	1.02
*Bos taurus*	0.66	0.82	1.32	1.32	0.96
*Mus musculus*	0.69	0.84	1.15	1.27	0.99
*Pongo abelii*	0.69	0.94	1.06	1.12	0.95

**Table 6:** (**a**) Frequency of tRNA genes in the selected mammalian species for the most preferentially used codons in the EPB41L3 gene in *Homo sapiens*. (**b**) Frequency of tRNA genes in the selected mammalian species for the most preferentially used codons in the EPB41L3 gene in *Pongo abelii*. (**c**) Frequency of tRNA genes in the selected mammalian species for the most preferentially used codons in the EPB41L3 gene in *Rattus norvegicus*. (**d**) Frequency of tRNA genes in the selected mammalian species for the most preferentially used codons in the EPB41L3 gene in *Mus musculus*. (**e**) Frequency of tRNA genes in the selected mammalian species for the most preferentially used codons in the EPB41L3 gene in *Bos taurus*. The tRNA population corresponding to most favored codon in the EPB41L3 gene is highlighted with red.

***Homo sapiens* (Human)**			
**Amino Acid**	**Most Preferred Codons in EPB41L3**	**Isotypes of tRNA in Human Cells**	**Total Count**
Ala (A)	GCC	AGC (22), GGC (0), CGC (4), TGC (8)	34
Gly (G)	GGG	ACC (0), GCC (14), CCC (5), TCC (9)	28
Pro (P)	CCA	AGG (9), GGG (0), CGG (4), TGG (7)	20
Thr (T)	ACC	AGT (9), GGT (0), CGT (5), TGT (6)	20
Val (V)	GTG	AAC (9), GAC (0), CAC (11), TAC (5)	25
Ser (S)	AGT	AGA (9), GGA (0), CGA (4), TGA (4), ACT (0), GCT (8)	25
Arg (R)	CGA	ACG (7), GCG (0), CCG (4), TCG (6), CCT (5), TCT (6)	28
Leu (L)	CTG	AAG (9), GAG (0), CAG (9), TAG (3), CAA (6), TAA (4)	31
Phe (F)	TTT	AAA (0), GAA (10)	10
Asn (N)	AAC	ATT (0), GTT (20)	20
Lys (K)	AAA	CTT (15), TTT (12)	27
Asp (D)	GAC	ATC (0), GTC (13)	13
Glu (E)	GAG	CTC (8), TTC (7)	15
His (H)	CAC	ATG (0), GTG (10)	10
Gln (Q)	CAG	CTG (13), TTG (6)	19
Ile (I)	ATC	AAT (14), GAT (3), TAT (5)	22
Tyr (Y)	TAC	ATA (0), GTA (13)	13
Cys (C)	TGT	ACA (0), GCA (29)	29
Trp (W)	TGG	CCA (7)	7
Met (M)	ATG	CAT (9/10)	19
(**a**)			
***Pongo abelii* (Sumatran Orangutan)**			
**Amino Acid**	**Most Preferred Codons in EPB41L3**	**Isotypes of tRNA in *Pongo abelii* Cells**	**Total Count**
Ala (A)	GCC/GCA	AGC (21), GGC (0), CGC (4), TGC (9)	34
Gly (G)	GGA/GGG	ACC (0), GCC (8), CCC (7), TCC (6)	21
Pro (P)	CCA	AGG (8), GGG (0), CGG (4), TGG (7)	19
Thr (T)	ACC	AGT (10), GGT (0), CGT (5), TGT (6)	21
Val (V)	GTG	AAC (10), GAC (0), CAC (11), TAC (6)	27
Ser (S)	TCA/TCT	AGA (9), GGA (0), CGA (4), TGA (4), ACT (0), GCT (8)	25
Arg (R)	CGA	ACG (7), GCG (0), CCG (4), TCG (6), CCT (5), TCT (6)	28
Leu (L)	CTG	AAG (8), GAG (0), CAG (6), TAG (3), CAA (5), TAA (5)	27
Phe (F)	TTT	AAA (0), GAA (8)	8
Asn (N)	AAC	ATT (0), GTT (22)	22
Lys (K)	AAA	CTT (14), TTT (14)	28
Asp (D)	GAC	ATC (0), GTC (9)	9
Glu (E)	GAG	CTC (5), TTC (14)	19
His (H)	CAC	ATG (0), GTG (12)	12
Gln (Q)	CAG	CTG (11), TTG (6)	17
Ile (I)	ATC	AAT (13), GAT (3), TAT (6)	22
Tyr (Y)	TAC	ATA (0), GTA (12)	12
Cys (C)	TGT	ACA (0), GCA (27)	27
Trp (W)	TGG	CCA (7)	7
Met (M)	ATG	CAT (8/10)	18
(**b**)			
***Rattus norvegicus* (Brown Rat)**			
**Amino Acid**	**Most Preferred Codons in EPB41L3**	**Isotypes of tRNA in *Rattus norvegicus* Cells**	**Total Count**
Ala (A)	GCC	AGC (31), GGC (0), CGC (3), TGC (7)	41
Gly (G)	GGA	ACC (0), GCC (11), CCC (5), TCC (9)	25
Pro (P)	CCA	AGG (8), GGG (0), CGG (3), TGG (6)	17
Thr (T)	ACC	AGT (7), GGT (0), CGT (4), TGT (5)	16
Val (V)	GTG	AAC (6), GAC (0), CAC (6), TAC (3)	15
Ser (S)	TCT	AGA (9), GGA (0), CGA (3), TGA (4), ACT (0), GCT (11)	27
Arg (R)	AGG	ACG (6), GCG (0), CCG (3), TCG (5), CCT (7), TCT (6)	27
Leu (L)	CTG	AAG (6), GAG (0), CAG (10), TAG (3), CAA (3), TAA (2)	24
Phe (F)	TTC	AAA (0), GAA (8)	8
Asn (N)	AAC	ATT (0), GTT (13)	13
Lys (K)	AAG	CTT (11), TTT (6)	17
Asp (D)	GAC	ATC (0), GTC (15)	15
Glu (E)	GAG	CTC (9), TTC (10)	19
His (H)	CAC	ATG (0), GTG (10)	10
Gln (Q)	CAG	CTG (10), TTG (5)	15
Ile (I)	ATC	AAT (8), GAT (0), TAT (3)	11
Tyr (Y)	TAC	ATA (0), GTA (3)	3
Cys (C)	TGC	ACA (0), GCA (40)	40
Trp (W)	TGG	CCA (7)	7
Met (M)	ATG	CAT (8/5)	13
(**c**)			
***Mus musculus* (House Mouse)**			
**Amino Acid**	**Most Preferred Codons in EPB41L3**	**Isotypes of tRNA in *Mus musculus* cells**	**Total Count**
Ala (A)	GCC	AGC (13), GGC (0), CGC (9), TGC (11)	33
Gly (G)	GGA	ACC (0), GCC (13), CCC (6), TCC (7)	26
Pro (P)	CCA	AGG (6), GGG (0), CGG (3), TGG (7)	16
Thr (T)	ACC	AGT (9), GGT (0), CGT (4), TGT (4)	17
Val (V)	GTG	AAC (7), GAC (0), CAC (10), TAC (3)	20
Ser (S)	TCT	AGA (8), GGA (0), CGA (3), TGA (3), ACT (0), GCT (7)	21
Arg (R)	CGC/CGG/AGG	ACG (6), GCG (0), CCG (3), TCG (5), CCT (5), TCT (5)	24
Leu (L)	CTG	AAG (5), GAG (0), CAG (10), TAG (3), CAA (4), TAA (4)	26
Phe (F)	TTT	AAA (0), GAA (7)	7
Asn (N)	AAC	ATT (0), GTT (13)	13
Lys (K)	AAG	CTT (19), TTT (13)	32
Asp (D)	GAC	ATC (0), GTC (16)	16
Glu (E)	GAG	CTC (11), TTC (8)	19
His (H)	CAC	ATG (0), GTG (10)	10
Gln (Q)	CAG	CTG (10), TTG (5)	15
Ile (I)	ATC	AAT (11), GAT (0), TAT (4)	15
Tyr (Y)	TAC	ATA (0), GTA (10)	10
Cys (C)	TGC	ACA (0), GCA (54)	54
Trp (W)	TGG	CCA (8)	8
Met (M)	ATG	CAT (9/8)	13
(**d**)			
***Bos taurus* (Aurochs/Domesticated Cattle)**			
**Amino Acid**	**Most Preferred Codons in EPB41L3**	**Isotypes of Trna in *Bos taurus* Cells**	**Total Count**
Ala (A)	GCC	AGC (28), GGC (0), CGC (8), TGC (14)	50
Gly (G)	GGC/GGG	ACC (0), GCC (13), CCC (14), TCC (7)	34
Pro (P)	CCG	AGG (12), GGG (0), CGG (4), TGG (8)	24
Thr (T)	ACC	AGT (12), GGT (0), CGT (4), TGT (8)	24
Val (V)	GTG	AAC (17), GAC (0), CAC (20), TAC (9)	46
Ser (S)	TCC	AGA (12), GGA (2), CGA (5), TGA (4), ACT (0), GCT (17)	40
Arg (R)	CGA	ACG (9), GCG (0), CCG (5), TCG (9), CCT (8), TCT (6)	37
Leu (L)	CTG	AAG (9), GAG (0), CAG (5), TAG (4), CAA (6), TAA (6)	30
Phe (F)	TTT/TTC	AAA (0), GAA (22)	22
Asn (N)	AAC	ATT (0), GTT (28)	28
Lys (K)	AAA	CTT (22), TTT (28)	50
Asp (D)	GAC	ATC (0), GTC (19)	19
Glu (E)	GAG	CTC (7), TTC (34)	41
His (H)	CAC	ATG (0), GTG (15)	15
Gln (Q)	CAG	CTG (22), TTG (7)	29
Ile (I)	ATC	AAT (17), GAT (0), TAT (5)	22
Tyr (Y)	TAC	ATA (0), GTA (16)	16
Cys (C)	TGT/TGC	ACA (0), GCA (27)	27
Trp (W)	TGG	CCA (8)	8
Met (M)	ATG	CAT (16/14)	30
(**e**)			

## Data Availability

Available data are presented in the manuscript.

## Supplementary Materials

The following are available online at https://www.mdpi.com/article/10.3390/cancers13112739/s1, Table S1: EPB41L3 gene coding sequence dataset, Table S2: Nucleotide composition of the EPB41L3 gene transcripts (A%, C% U%, and G%) with nucleotides at the third position (A3%, C3% U3%, and G3%), overall AU%, GC%, and AU3% composition along with GC12, GC3%, ENc, and CAI analysis.
