# Supplementary material for: Insight into Codon Utilization Pattern of Tumor Suppressor Gene EPB41L3 from Different Mammalian Species Indicates Dominant Role of Selection Force
Source: Cancers (Basel). 2021 Jun 1;13(11):2739. doi: 10.3390/cancers13112739 (PMC8198080; doi:10.3390/cancers13112739)
Supplement: Supplementary file 1 [file cancers-13-02739-s001.zip › cancers-1182879-supplementary.pdf]

cancers

MDPI

Supplementary Materials

Insight into Codon Utilization Pattern of Tumor Suppressor Gene EPB41L3 from Different Mammalian Species Indicates Dominant Role of Selection Force

Utsang Kumar [1], Rekha Khandia [1,*], Shailja Singhal [1], Nidhi Puranik [1], Meghna Tripathi [2], Atul Kumar Pateriya [2], Raju Khan [3], Talha Bin Emran [4], Kuldeep Dhama [5,*], Ashok Munjal [1], Taha Alqahtani [6] and Ali M. Alqahtani [6]

Table S1. EPB41L3 gene coding sequence dataset.

SL. No.	Species	Sequences/Parameters	Length (Base pairs)
1	*Homo sapiens*	NM_001384704.1	2307
2		NM_001384692.1	2622
3		NM_001384685.1	3318
4		NM_001384691.1	2634
5		NM_001384696.1	2544
6		NM_001281533.2	2652
7		NM_001384682.1	2652
8		NM_001384703.1	2325
9		NM_001384697.1	2517
10		NM_001384699.1	2394
11		NM_001384706.1	2253
12		NM_001384684.1	2757
13		NM_001384693.1	2610
14		NM_001384698.1	2430
15		NM_001384705.1	2295
16		NM_001384695.1	2553
17		NM_001384688.1	2703
18		NM_001281534.3	2598
19		NM_001384700.1	2376
20		NM_001384689.1	2676
21		NM_001330557.2	2757
22		NM_001384690.1	2676
23		NM_001384686.1	2721
24		NM_001384687.1	2703
25		NM_001384694.1	2598
26		NM_001384683.1	2271
27		NM_001384702.1	2340
28		NM_001384701.1	2349
29		NM_012307.5	3264
30		NM_001281535.2	2271
31	*Rattus norvegicus*	NM_053927.1	3318
32	*Bos taurus*	NM_001206169.1	3288
33	*Mus musculus*	NM_013813.2	2790
34	*Pongo abelii*	NM_001133169.1	2430

Table S2. Nucleotides composition of EPB41L3 gene transcripts (A%, C% U%, and G%) with nucleotides at third position (A3%, C3% U3%, and G3%), overall AU%, GC%, and AU3% composition along with GC12, GC3%, ENc, and CAI analysis.

Coding Sequences (CDSs)/ Accession number (EPB41L3)	Length	A%	C%	U%	G%	A3%	C3%	U3%	G3%	AU%	GC%	GC1	GC2	GC12	AU3%	GC3%	ENc	CAI
NM_001384704.1	2307	30.78	22.8	19.59	26.83	26.53	23.93	20.94	28.61	50.37	49.6	55.3	41.1	48.2	47.46	52.5	57.2	0.775
NM_001384692.1	2622	29.9	23.61	19.15	27.35	25.51	23.68	20.37	30.43	49.05	51	56.3	42.4	49.35	45.88	54.1	58.4	0.771
NM_001384685.1	3318	28.12	25.32	20.25	26.31	23.6	26.22	20.98	29.2	48.37	51.6	56.1	43.4	49.75	44.58	55.4	57.6	0.778
NM_001384691.1	2634	30.26	23.46	18.83	27.45	25.51	23.8	20.16	30.52	49.09	50.9	56.6	41.8	49.2	45.67	54.3	57.9	0.773
NM_001384696.1	2544	30.07	23.62	18.83	27.48	25.35	24.17	20.05	30.42	48.9	51.1	56.5	42.2	49.35	45.4	54.6	57.7	0.773
NM_001281533.2	2652	30.02	23.34	19.42	27.22	25.45	23.64	20.81	30.09	49.43	50.6	55.5	42.4	48.95	46.27	53.7	58.6	0.77
NM_001384682.1	2652	30.02	23.34	19.42	27.22	25.45	23.64	20.81	30.09	49.43	50.6	55.5	42.4	48.95	46.27	53.7	58.6	0.77
NM_001384703.1	2325	30.49	22.67	20.26	26.58	26.45	23.74	21.68	28.13	50.75	49.2	54.1	41.8	47.95	48.13	51.9	58.3	0.771
NM_001384697.1	2517	30.08	23.68	19.03	27.21	25.51	23.96	20.26	30.27	49.11	50.9	56.3	42.2	49.25	45.77	54.2	57.7	0.773
NM_001384699.1	2394	30.62	22.64	20.18	26.57	26.69	23.56	21.68	28.07	50.79	49.2	54.3	41.7	48	48.37	51.6	57.8	0.773
NM_001384706.1	2253	30.63	22.95	19.44	26.99	26.36	24.23	20.64	28.76	50.07	49.9	55.5	41.3	48.4	47	53	57.1	0.775
NM_001384684.1	2757	30.18	23.29	19.22	27.31	25.68	23.39	20.67	30.25	49.4	50.6	56	42.1	49.05	46.35	53.6	58.4	0.771
NM_001384693.1	2610	30.19	23.45	19	27.36	25.4	23.91	20.46	30.23	49.2	50.8	56.2	42.1	49.15	45.86	54.1	57.7	0.773
NM_001384698.1	2430	30.66	22.63	20	26.71	26.67	23.46	21.48	28.4	50.66	49.3	54.7	41.5	48.1	48.15	51.9	58	0.773
NM_001384705.1	2295	30.37	22.96	19.96	26.71	26.54	23.79	21.18	28.5	50.33	49.7	54.9	41.8	48.35	47.71	52.3	58.1	0.773
NM_001384695.1	2553	30.12	23.66	18.88	27.34	25.5	23.85	20.09	30.55	49	51	56.6	42	49.3	45.59	54.4	57.9	0.773
NM_001384688.1	2703	30.04	23.42	19.09	27.45	25.53	23.64	20.42	30.41	49.13	50.9	56.3	42.3	49.3	45.95	54.1	58.4	0.771
NM_001281534.3	2598	30.22	23.48	18.98	27.33	25.52	23.9	20.32	30.25	49.19	50.8	56.2	42	49.1	45.84	54.2	57.7	0.773
NM_001384700.1	2376	30.51	22.77	19.87	26.85	26.52	23.74	21.21	28.54	50.38	49.6	54.9	41.7	48.3	47.73	52.3	58.1	0.773
NM_001384689.1	2676	30.04	23.47	19.28	27.2	25.67	23.43	20.63	30.27	49.33	50.7	56.1	42.3	49.2	46.3	53.7	58.4	0.771
NM_001330557.2	2757	30.18	23.29	19.22	27.31	25.68	23.39	20.67	30.25	49.4	50.6	56	42.1	49.05	46.35	53.6	58.4	0.771
NM_001384690.1	2676	30.04	23.47	19.28	27.2	25.67	23.43	20.63	30.27	49.33	50.7	56.1	42.3	49.2	46.3	53.7	58.4	0.771
NM_001384686.1	2721	30.14	23.3	19.37	27.2	25.69	23.48	20.84	29.99	49.5	50.5	55.7	42.3	49	46.53	53.5	58.2	0.771
NM_001384	2703	30.04	23.42	19.09	27.45	25.53	23.64	20.42	30.41	49.13	50.9	56.3	42.3	49.3	45.95	54.1	58.4	0.771

687.1																		
NM_001384694.1	2598	29.87	23.48	19.28	27.37	25.29	23.9	20.55	30.25	49.15	50.8	55.8	42.6	49.2	45.84	54.2	58.7	0.77
NM_001384683.1	2271	30.74	22.81	19.77	26.68	26.55	24.04	21.14	28.27	50.51	49.5	54.8	41.3	48.05	47.69	52.3	57	0.776
NM_001384702.1	2340	30.47	22.78	20.04	26.71	26.54	23.85	21.41	28.21	50.51	49.5	54.5	41.9	48.2	47.95	52.1	57.8	0.774
NM_001384701.1	2349	30.52	22.82	20.09	26.56	26.69	23.5	21.46	28.35	50.62	49.4	54.7	41.6	48.15	48.15	51.9	58.1	0.773
NM_012307.5	3264	27.97	25.46	20.16	26.41	23.44	26.47	20.77	29.32	48.13	51.9	56.2	43.6	49.9	44.21	55.8	57.6	0.778
NM_001281535.2	2271	30.74	22.81	19.77	26.68	26.55	24.04	21.14	28.27	50.51	49.5	54.8	41.3	48.05	47.69	52.3	57	0.776
NM_053927.1	3318	27.55	26.58	19.14	26.73	22.06	28.57	18.72	30.65	46.68	53.3	57.5	43.2	50.35	40.78	59.2	53.7	0.765
NM_001206169.1	3288	26.67	27.65	18.25	27.43	19.8	31.48	17.06	31.66	44.92	55.1	58.6	43.5	51.05	36.86	63.1	54.7	0.74
NM_013813.2	2790	29.35	24.37	18.24	28.03	23.33	25.05	19.35	32.26	47.6	52.4	57	42.9	49.95	42.69	57.3	54.8	0.781
NM_001133169.1	2430	30.82	22.72	19.96	26.5	27.04	23.83	21.23	27.9	50.78	49.2	54.4	41.5	47.95	48.27	51.7	57.9	0.818
**Average Value ±SD**	**2617.412 ± 299.473**	**29.954 ± 0.955**	**23.574 ± 1.115**	**19.422 ± 0.537**	**27.051 ± 0.400**	**25.45 ± 1.474**	**24.304 ± 1.645**	**20.595 ± 0.877**	**29.649 ± 1.114**	**49.375 ± 1.231**	**50.626 ± 1.237**	**55.774 ± 0.971**	**42.144 ± 0.627**	**48.959 ± 0.741**	**46.045 ± 2.281**	**53.953 ± 2.271**	**57.656 ± 1.132**	**0.773 ± 010**
